# Change in beak overhangs of cliff swallows over 40 years: Partly a response to parasites?

**DOI:** 10.1371/journal.pone.0263422

**Published:** 2022-02-22

**Authors:** Gigi S. Wagnon, Olivia M. Pletcher, Charles R. Brown

**Affiliations:** Department of Biological Sciences, University of Tulsa, Tulsa, Oklahoma, United States of America; CNRS: BIOM Integrative Biology of Marine Organisms, FRANCE

## Abstract

Some birds exhibit a maxillary overhang, in which the tip of the upper beak projects beyond the lower mandible and may curve downward. The overhang is thought to help control ectoparasites on the feathers. Little is known about the extent to which the maxillary overhang varies spatially or temporally within populations of the same species. The colonial cliff swallow (*Petrochelidon pyrrhonota*) has relatively recently shifted to almost exclusive use of artificial structures such as bridges and highway culverts for nesting and consequently has been exposed to higher levels of parasitism than on its ancestral cliff nesting sites. We examined whether increased ectoparasitism may have favored recent changes in the extent of the maxillary overhang. Using a specimen collection of cliff swallows from western Nebraska, USA, spanning 40 years and field data on live birds, we found that the extent of the maxillary overhang increased across years in a nonlinear way, peaking in the late 2000’s, and varied inversely with cliff swallow colony size for unknown reasons. The number of fleas on nestling cliff swallows declined in general over this period. Those birds with perceptible overhangs had fewer swallow bugs on the outside of their nest, but they did not have higher nesting success than birds with no overhangs. The intraspecific variation in the maxillary overhang in cliff swallows was partly consistent with it having a functional role in combatting ectoparasites. The temporal increase in the extent of the overhang may be a response by cliff swallows to their relatively recent increased exposure to parasitism. Our results demonstrate that this avian morphological trait can change rapidly over time.

## Introduction

Beaks in some species of birds have a maxillary overhang, in which the upper mandible is longer than the lower and may curve downward to result in a slight hook [1]. The overhang is composed of keratin layers within the rhinotheca that cover the bone of the beak, with the rhinotheca near the beak tip subject to wear and growing more rapidly than the less distal rhinotheca layers [2]. The functional significance of the overhang has attracted surprisingly little attention. While the extent of the beak’s hook may be important in foraging in some species [3, 4], the maxillary overhang has been primarily studied as an anti-parasite adaptation [1, 5–7]. During preening the lower mandible creates a shearing force against the overhang, serving to damage ectoparasites on the feathers and leading to lowered parasitism on the body [1]. Experimental reduction in beak overhangs of rock pigeons (*Columba livia*) led to increases in feather lice [6], suggesting that the overhang has an important anti-parasite function. However, some studies have shown that relatively long maxillary overhangs can also be detrimental to ectoparasite control [7–9], suggesting that an intermediate degree of overhang may confer the greatest advantage during preening.

Little is known about the extent of intraspecific variation in the maxillary overhang or how parasitism may have led to selection on this component of avian beak morphology. Here we examine variation in the maxillary overhangs of cliff swallows (*Petrochelidon pyrrhonota*) and assess whether the observed patterns are consistent with those expected if the overhang functions in parasite removal. Cliff swallows are highly colonial insectivores that are subject to parasitism by fleas, lice, mites, and hematophagous bugs [10–12], and some of these parasites have detrimental effects on the birds’ annual survival and nesting success [10, 11, 13–18]. Within the last 50 years, the cliff swallow has shifted its nesting almost exclusively to artificial sites such as bridges and highway culverts, and the microclimate and nest stability of these artificial sites have led to greater exposure to parasitism than what the birds experienced on natural cliff nesting sites [18–21]. As in other highly social species [22–25], parasitism by hematophagous bugs and fleas in cliff swallows tends to increase with colony size [11, 13]. The prevalence of parasites and the fitness costs associated with them suggest that the maxillary overhang in cliff swallows is a potential anti-parasite adaptation that might vary both temporally and spatially in response to the greater exposure to parasites the birds have encountered in recent years and in the large colonies many occupy.

In this study we examine maxillary overhangs within a population of cliff swallows that we have studied for 40 years in western Nebraska. If the maxillary overhang helps in controlling parasites, we make the following specific predictions. (1) The presence of the maxillary overhang in cliff swallows should have increased over time in response to their recent greater exposure to parasitism, which in our study area began when the birds switched heavily to artificial nesting sites in the 1980’s [19]. (2) Because cliff swallows show some phenotypic specialization for colony size [26, 27], individuals occupying the larger colonies that have more parasites should have greater maxillary overhangs in general than those birds using smaller colonies. (3) An increase in the average extent of the overhang should lead to fewer (or at least no change) in parasites now compared to the 1980’s despite the potential for greater current exposure to parasites. (4) Cliff swallows with maxillary overhangs should have fewer parasites than those without perceptible overhangs, as found in other species [4–9]. (5) Anti-parasite advantages should lead to birds with more perceptible overhangs having greater reproductive success than those without such overhangs.

We use two kinds of data in this study: a museum collection of over 1100 cliff swallows collected opportunistically over 40 years in our study area to test predictions (1) and (2), and counts of parasites on nests and observations of live birds to test predictions (3)-(5). Given that previous work on the functional significance of the maxillary overhang in other species has involved primarily laboratory experiments or comparisons among different populations that may also be subject to resource-related selection on bill morphology, our study is unique in examining temporal and spatial correlates of maxillary overhang within a single population.

## Methods

### Study organisms and study site

**Fig 1:**
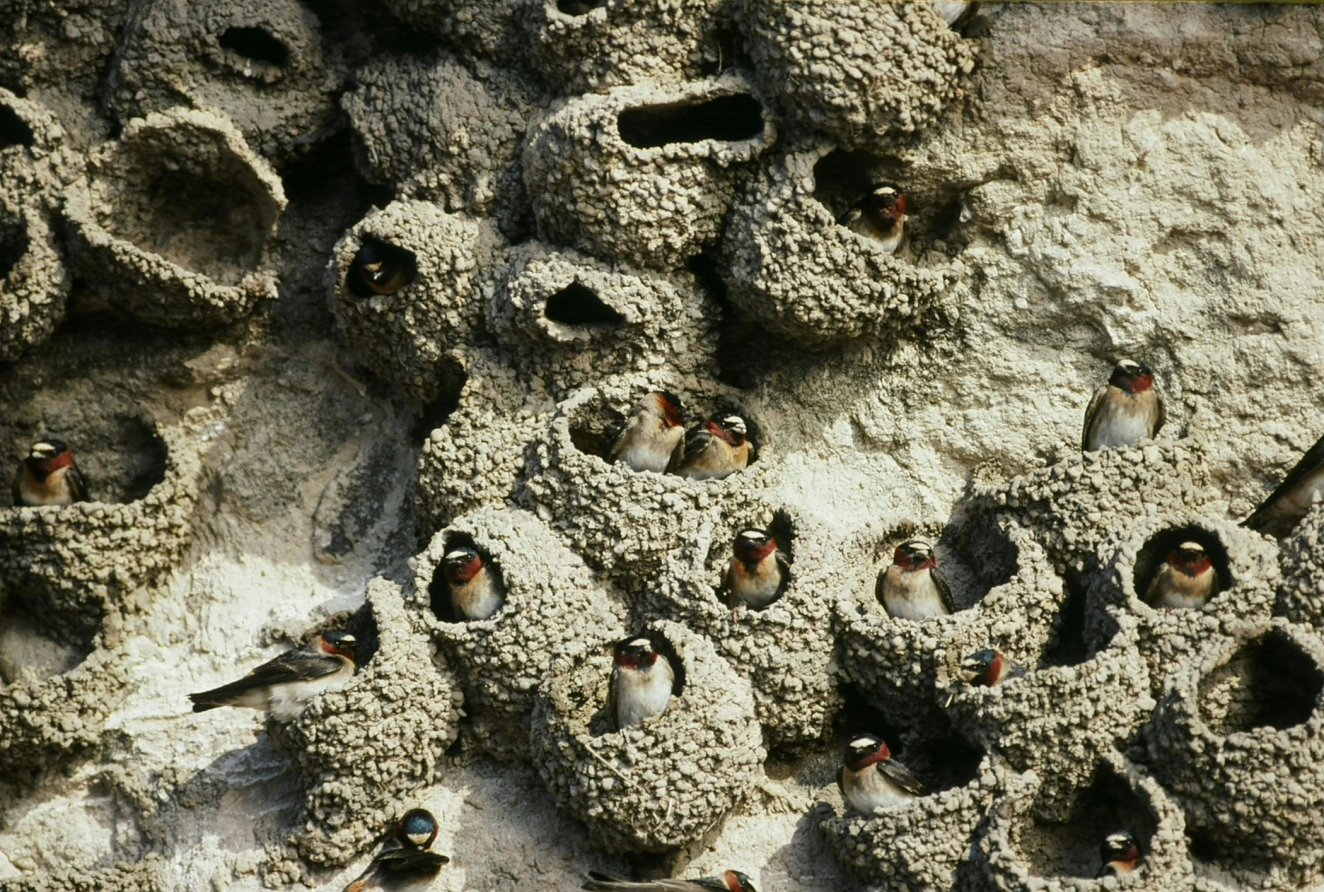
Cliff swallows at a nesting colony in western Nebraska.

The cliff swallow is a highly colonial passerine that breeds commonly throughout the western half of North America and less commonly eastward [21]. In its original habitat, the species built its gourd-shaped mud nests underneath horizontal overhangs on the sides of steep cliffs, often in dense clusters (Fig 1), but now many cliff swallows nest under the sides of bridges and buildings or inside concrete culverts underneath roads [28]. These birds winter in southern South America, primarily Argentina [21]. Cliff swallows feed on a wide array of insect taxa [11] that are caught in flight.

Parasites of cliff swallows in our study area include swallow bugs (Hemiptera: Cimicidae: *Cimex vicarius*), fleas (Siphonaptera: Ceratophyllidae: *Ceratophyllus celsus*), mites (Astigmata: Avenzoariidae: *Pteronyssoides obscurus*), and two species of feather lice (Ischnocera: Philopteridae: *Acronirmus* [formerly *Brueelia*; 29] *longa* and Amblycera: Menoponidae: *Machaerilaemus malleus*) [10–12]. The hematophagous bugs and fleas are the most numerous and have the greatest effects on cliff swallows by reducing survival of nestlings and adults, affecting feather asymmetry and site use, and constraining the duration of the nesting season [11, 13–17, 30]. Lice are also associated with a reduction in annual survival of adult cliff swallows [10].

We studied cliff swallows near the Cedar Point Biological Station (41.2097° N, 101.6480° W) in western Nebraska, USA, along the North and South Platte rivers. The study area includes portions of Keith, Garden, Deuel, Lincoln, and Morrill counties. Our work was done primarily at cliff swallow colonies on highway bridges and box-shaped culverts underneath roads or railroad tracks [28]. Colonies were defined as birds from groups of nests that interacted at least occasionally in defense against predators or by sharing information on the whereabouts of food [11]. Typically, all the nests on a given bridge or culvert constituted a colony, and most colonies were at least 0.5 km from the next nearest. Colony size varied widely, ranging from 2 to 6000 nests (mean ± SE = 404 ± 11 nests, *n* = 3277 colonies), with some birds also nesting solitarily.

### Specimen collection

Cliff swallows were collected opportunistically in 1982–2021 whenever salvageable specimens were found in the course of our research, and preserved as skins [31]. These included birds dying in mist-netting accidents, on roads due to collisions with vehicles, during severe weather events, due to other miscellaneous causes (e.g., drowning during fights, nest falls, killed by predators), or for unknown reasons. The colony at which a dead bird was found was designated as the colony size for that specimen, as banded birds found dead were invariably at the site where they were known to be resident. Colony size refers to the number of active nests at a site that year, and was determined from active-nest counts or estimation from the number of birds present [11, 28]. For any colony where we had more than 50 specimens in a year, we randomly selected 50 from each site for this study. We scored maxillary overhangs of 1207 cliff swallow specimens from a total of 230 colonies across the 40 years; of these, 1108 had full information on colony size, sex, and other variables. Only adult birds (ones at least one year old, known from plumage) were included in this study. We noted which birds were from colonies where parasites had been removed by fumigation [11, 13] and accounted for colony fumigation status in the analyses. All specimens were from the collection at the University of Tulsa, except for 9 specimens collected in the study area in 1984 from the collection of the American Museum of Natural History and 8 specimens collected in the study area in 1985 from the collection of the Peabody Museum of Natural History.

### Scoring maxillary overhang

**Fig 2:**
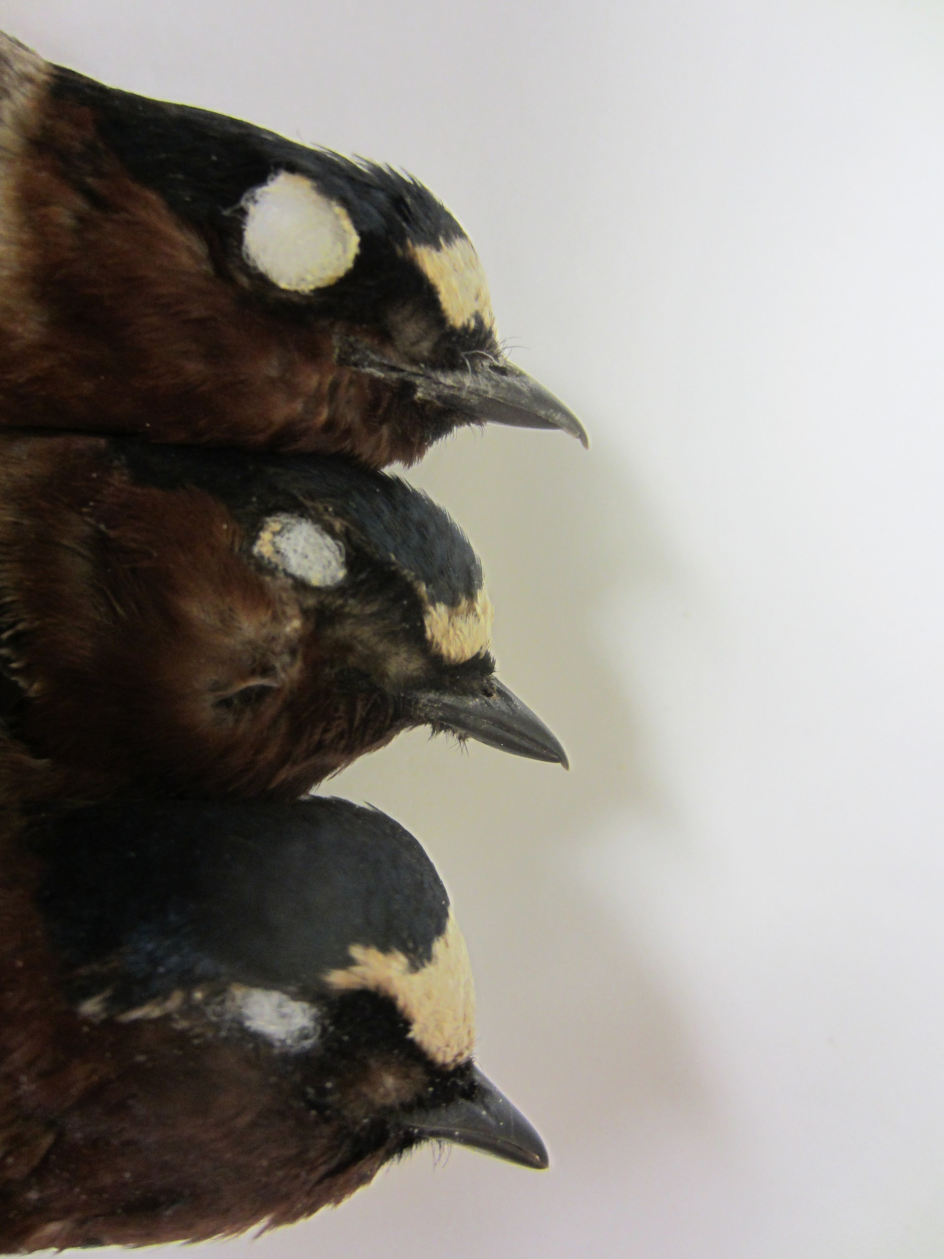
Examples of the three categories of maxillary overhang used for scoring cliff swallow specimens: 0 (bottom), 1 (middle), and 2 (top).

Each specimen was assigned to one of three categories (Fig 2). Birds with type 0 had no perceptible overhang; those with type 2 had a noticeable downward curving of the upper mandible; and birds with an overhang intermediate between these were type 1 (Fig 2). Repeatability of scoring was done for a random sample of 50 birds that were re-scored 3 months later while blind to the previous measures. To account for possible relationships between beak overhang and beak size or overall head size, the beak width at its widest point at the cere was measured with calipers, and (for birds collected in 1982–2018) the head size was measured and converted to volume by G.S.W., as described in Wagnon and Brown [31]. The wing length of the unflattened wing from the shoulder to the tip of the longest primary was also measured. For birds from 1982–2018, all scoring of overhangs and measurements were done by G.S.W. and those from 2019–2021 by C.R.B. Results were almost identical for both the entire dataset and for those measured only by G.S.W., so we assume no systematic bias in the measurements between the two people. The measurements for repeatability were done by G.S.W.

Maxillary overhangs in live cliff swallows were scored at five non-fumigated colonies in 2020–21 where we also did regular nest checks. We used a 20-60X spotting scope to observe birds sitting in their nest entrances. Observations were made at each site while the colony was primarily incubating, which was a time that most nest intrusions by neighbors or non-residents had largely ceased and thus we were mostly seeing actual residents at the nests [11]. However, because birds were not color-marked, we scored nests as either (1) having at least one resident with a visible beak overhang (corresponding to beak score 2 for the specimens) or (2) as having only birds with no perceptible overhang. This latter group included birds scored as 0 and 1 on specimens because the smaller overhangs were difficult to visually distinguish reliably on live birds under field conditions. Because some nests scored as no overhang may have had a resident with a type 2 overhang that was overlooked, our tests for differences among nests with owners of these overhang types were conservative. All observations and overhang scoring of live birds were done by one person (O.M.P.).

### Ethical note

Specimens were salvaged under authority of the Bird Banding Laboratory of the United States Geological Survey (permit 20948) and a series of Scientific Permits from the Nebraska Game and Parks Commission. All animal use was approved by a series of protocols from the Institutional Animal Care and Use Committees of Yale University and the University of Tulsa. Field sites were on public right-of-way, requiring no permission to access, or on private land where landowners had given permission for entry.

### Parasite counts and nesting success

Because fleas are active on the outsides of cliff swallow nests only for a brief time early in the spring [11], we assessed flea parasitism for nests by removing nestling cliff swallows and scoring the number of fleas crawling on the nestlings’ bodies [13]. For the flea analysis, we used data from colonies throughout the study area in 1982–1989 and 2015–2018 where nestlings were removed at 10 days of age and the number of *Ceratophyllus* fleas counted [11, 13]. Cliff swallow nests were checked (using a flashlight and dental mirror) at 2–4 day intervals at colonies, allowing us to know hatching date and nestling age for each nest. Flea counts were done the same way each year throughout the study, with C.R.B. training and supervising the bird handling and parasite counts each year. We also recorded brood size and nestling weight at the time fleas were counted and knew the hatching date and colony size. Data were available for 4453 nestlings from a total of 58 colonies in 1982–1989 and 2015–2018 [18]. Only non-fumigated nests were used in analysis of fleas.

Nests where birds were observed in 2020–21 were checked to record nest contents. For these nests, at the time of each nest check we estimated the number of ectoparasitic swallow bugs visible on the outside of each nest. This provides a relative measure of the extent of swallow bug parasitism per nest [32] and is the best way known to score bug parasitism at active nests. One person (O.M.P.) did all swallow bug counts on nests, with the scoring of maxillary overhangs of nest owners done without knowledge of the parasite counts or eventual success of a nest. Although we had bug counts per nest taken throughout the nesting season, for this study we used only the last count prior to when the eggs in a nest hatched. This standardized the counts with respect to host nesting stage for each nest, and also meant that we had bug numbers for nests that failed soon after hatching (which typically led to a major reduction in the number of bugs present after failure). Nesting success was measured as the number of nestlings still alive 17 days after hatching. Nests failing prior to hatching or before 17 days were scored as having 0 nestlings surviving. We had 190 nests from 2020–21 with information on owners’ maxillary overhang, swallow bug parasitism, and nesting success.

### Statistical analyses

Analyses of variables predicting maxillary overhang in specimens, nesting success and bug parasitism in live birds, and the number of fleas per nestling used mixed-model regression implemented with Proc MIXED in SAS [33]. Independent covariates (fixed effects) were identified *a priori* based on the questions posed here or past work (Table 1). Interactions between fixed effects were explored in preliminary analyses, but none was significant and thus not presented here. In analyzing fleas per nestling, we treated year as a categorical predictor variable (e.g., two categories, 1982–89 and 2015–18), designated as decade, given that a ~25-year gap existed in when these data were collected. Analysis of the specimen collection, in which specimens were collected continuously across the entire time of the study, treated year as a continuous predictor variable. Fumigation status of a colony site was a categorical (yes/no) variable. Overhang type was an ordinal response variable (0, 1, 2), and because categorizing the overhangs on the specimens was generally very obvious (Fig 2), we considered the intervals among them equivalent, allowing overhang type to be treated as a continuous variable in analyses [34].

**Table 1:** Mixed-model results showing fixed-effect and random-effect predictors of beak overhang score (0, 1, 2) in cliff swallow specimens (*N* = 1108).

Fixed effect	β	SE	*F*	df	*P*
year	6.9396	1.1563	36.02	1, 900	< 0.0001
year*year	‒0.00173	0.000289	35.90	1, 900	< 0.0001
colony size	‒0.00016	0.000054	9.04	1, 900	0.0027
bill width	0.8261	0.6208	1.77	1, 900	0.18
wing length	0.1527	0.1839	0.69	1, 900	0.41
colony fumigation status^1^	‒0.05575	0.07440	0.56	1, 900	0.45
sex^2^	0.01658	0.03701	0.20	1, 900	0.65
Random effect	Estimated variance component	SE	Level	*Z*	*P*
colony-site-by-year	0.05003	0.01857	230	2.69	0.0035
colony site	0.000248	0.01006	78	0.02	0.49

^1^Relative to fumigation = yes as baseline.

^2^Relative to male as baseline.

To account for non-independence of observations (and potential pseudoreplication) in our data, in the mixed models we used the following random intercept variables where appropriate: colony site, coded as the same site designation across years, to account for potential spatial dependence of a colony site’s physical location; colony-site-by-year, coded the same for all observations at a colony site in the same year but different between years, to account for dependence among observations at a single colony within a year; and (for the flea analysis) nest identity, coded the same for all nestlings within the same nest in a given year but different among years, to account for potential dependence among nestlings from the same nest.

Repeatability in scoring maxillary overhang by G.S.W. was assessed with the intraclass correlation coefficient [35], calculated from a model with specimen number as the independent predictor of maxillary overhang and using Proc GLM in SAS.

## Results

### Overhang variation among specimens

Repeatability of overhang scoring on specimens was high and significant (*r*_*I*_ = 0.751, *F*_1,49_ = 7.16, *P* < 0.0001). The two significant predictors of the extent of maxillary overhang were year and colony size (Table 1). There was no significant effect of sex, wing length, bill width, or whether a colony site was fumigated while controlling for colony site and colony-site-by-year as random effects (Table 1). For the subset of 1043 specimens from 1992–2018, there was no significant effect of head volume (β = 0.0698, SE = 0.1600, *F*_1,833_ = 0.03, *P* = 0.86) in a separate analysis that was otherwise identical to that in Table 1.

**Fig 3:**
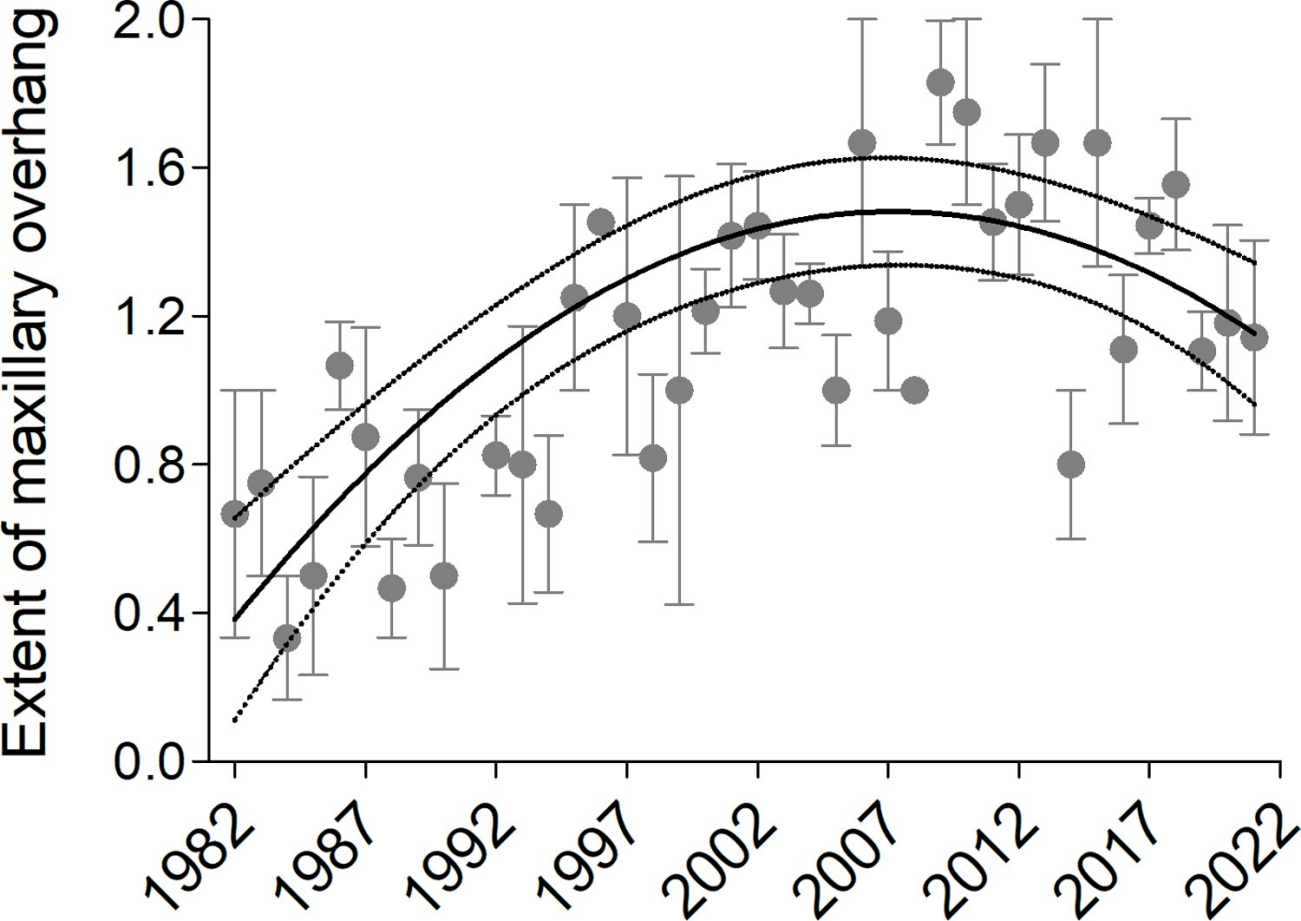
Extent of the maxillary overhang in cliff swallow specimens in relation to year, 1982–2021. Yearly means (± 1 SE) are shown with gray dots and bars. The predicted values from a mixed model regression (Table 1) with other variables held at their mean are shown with a solid line, and the 95% confidence interval of the predicted values are shown with dotted lines.

Maxillary overhang varied with year in a curvilinear pattern (Table 1), seemingly increasing from 1982 until about 2009 and declining afterwards (Fig 3). A model with a nonlinear effect of year was a better fit (AIC = 2121.8) than an otherwise identical one with only a linear effect of year (AIC = 2137.1).

**Fig 4:**
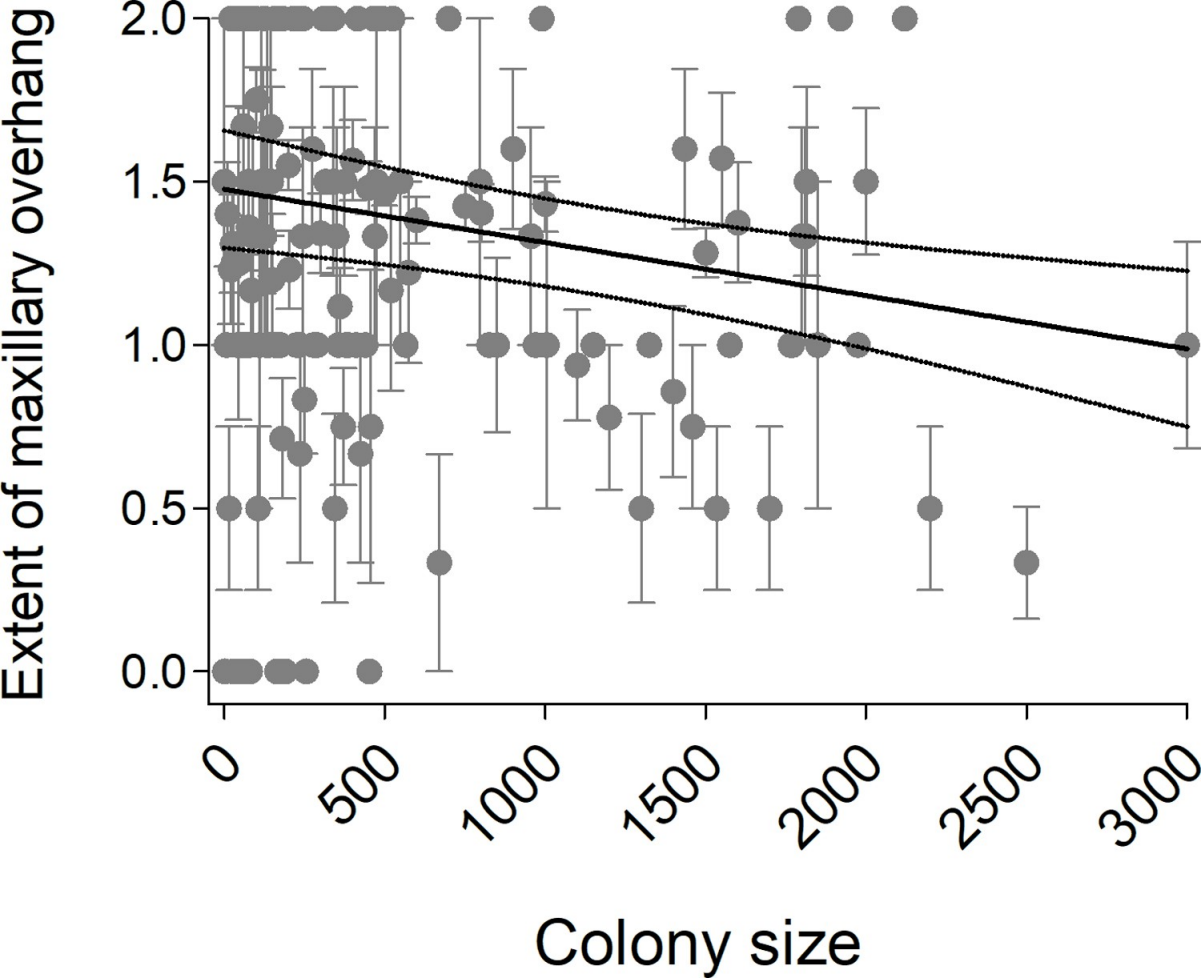
Extent of the maxillary overhang in cliff swallow specimens in relation to colony size (no. active nests at a site). Colony-size means (± 1 SE) are shown with gray dots and bars. The predicted values from a mixed model regression (Table 1) with other variables held at their mean are shown with a solid line, and the 95% confidence interval of the predicted values are shown with dotted lines.

The extent of the maxillary overhang declined with increasing colony size (Fig 4, Table 1).

### Changes in fleas over time

The mean (± SE) number of fleas counted on cliff swallow nestlings in the 1980’s, 0.896 (± 0.0329, *n* = 3020), was about twice that in the 2010’s, 0.456 (± 0.0304, *n* = 1434). Decade was a significant predictor of flea count (*F*_1,3099_ = 8.63, *P* = 0.0033), while controlling for the fixed effects of brood size (*F*_1,3099_ = 22.38, *P* < 0.0001), hatching date (*F*_1,3099_ = 6.83, *P* = 0.0090), body mass (*F*_1,3099_ = 19.16, *P* < 0.0001), and colony size (*F*_1,3099_ = 0.71, *P* = 0.40) and the random effects of colony site (*Z* = 1.35, *P* = 0.089), colony-site-by year (*Z* = 1.69, *P* = 0.046), and nest identity (*Z* = 12.81, *P* < 0.0001).

### Overhangs in relation to swallow bug parasitism and nesting success

**Fig 5:**
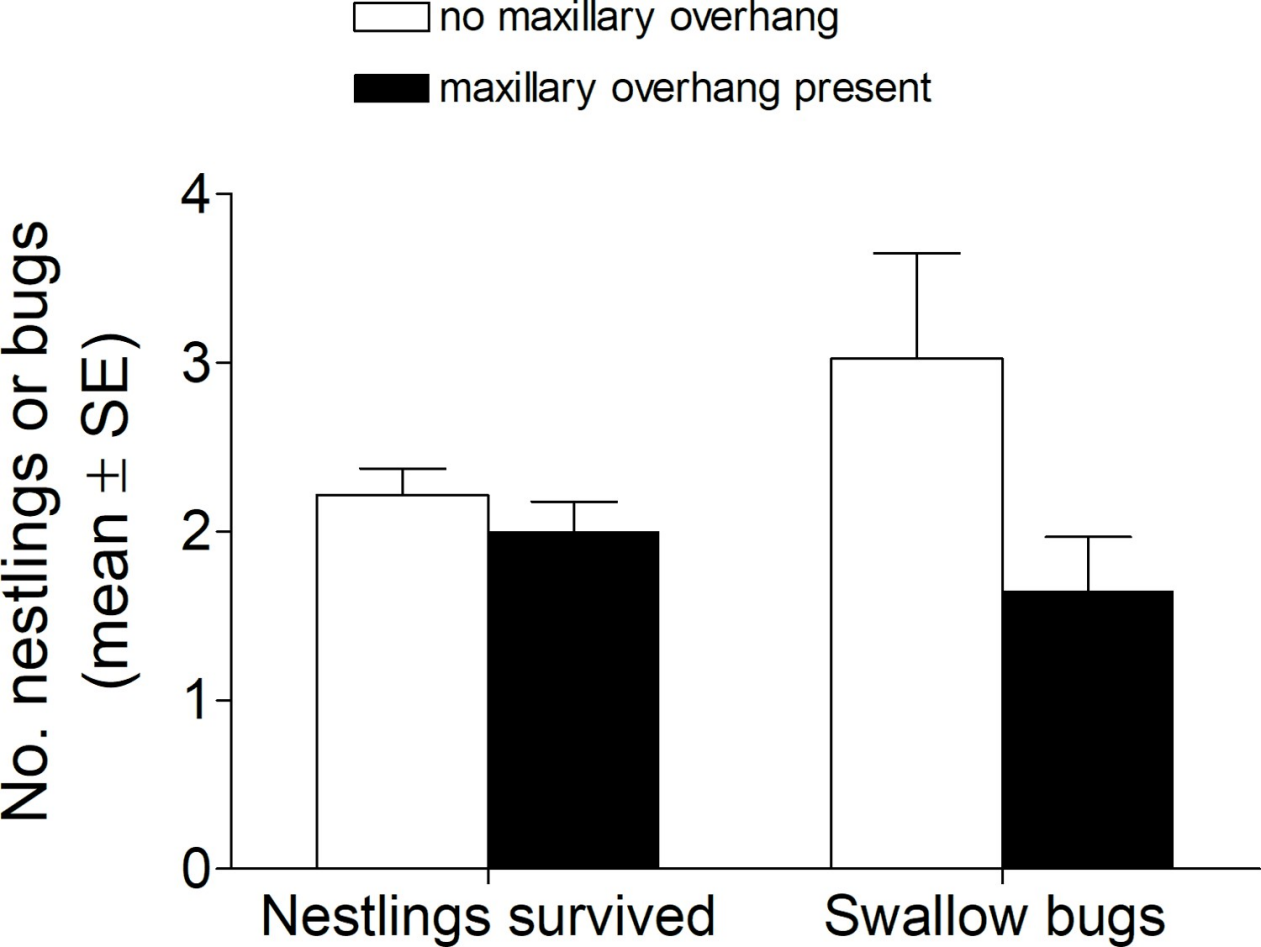
Mean (± SE) number of nestling cliff swallows surviving to day 17 per nest and number of swallow bugs counted per nest where at least one nest owner had a perceptible maxillary overhang (dark bars; *N* = 75 nests) and where no nest owners had perceptible overhangs (light bars; *N* = 115). The number of bugs differed significantly among nest types but the number of nestlings surviving did not (see text).

The number of swallow bugs counted on the outside of the nest was significantly higher for cliff swallow nests where owners had no perceptible maxillary overhang than at nests where at least one owner had an overhang (Fig 5, *F*_1,183_ = 4.11, *P* = 0.044); laying date had no effect on bugs (*F*_1,183_ = 1.71, *P* = 0.19) while controlling for colony site as a random effect (*Z* = 1.01, *P* = 0.15). Nest success (the number of nestlings surviving to day 17) where no owner had a perceptible maxillary overhang was not significantly different from nests where at least one owner had an overhang (Fig 5, *F*_1,181_ = 0.79, *P* = 0.38); nest success was significantly affected by the number of swallow bugs (*F*_1,181_ = 8.01, *P* = 0.005), clutch size (*F*_1,181_ = 5.29, *P* = 0.022), and laying date (*F*_1,181_ = 12.60, *P* = 0.0005) while controlling for colony site as a random effect (*Z* = 1.29, *P* = 0.10).

## Discussion

Our analyses show that the extent of the maxillary overhang of cliff swallows in western Nebraska increased over the period 1982–2021 but in a nonlinear way, seeming to peak in the late 2000’s and then declining. The extent of the maxillary overhang was greater among birds in smaller colonies. Accompanying the temporal increase in the overhang was a reduction in the number of fleas on nestlings in general during the same time period. Birds with perceptible overhangs had fewer swallow bugs on the outside of their nest, but this reduction in swallow bug parasitism did not translate into higher nesting success for cliff swallows with more visible overhangs. We found no evidence that the extent of the maxillary overhang varied systematically with other aspects of cliff swallow morphology, with non-significant effects of wing length, beak width, and head size, and sex had no effect on the extent of the overhang.

Cliff swallows have only relatively recently come into contact with more parasites by moving off of natural cliff nesting sites and onto artificial sites such as bridges where parasite survival is likely enhanced [18, 20]. Given the birds’ recent exposure to higher levels of parasitism (and assuming that the maxillary overhang can be help control ectoparasites on a cliff swallow’s feathers; see below), we predicted a temporal response in the extent of the maxillary overhang in this population. The increase in maxillary overhang in the immediate aftermath of the 1980’s nesting-site shift supports this prediction (Fig 3). Interestingly, the curvilinear pattern (Fig 3) is consistent with other work showing that maxillary overhangs that are too long can be counterproductive in parasite removal [7–9]; long overhangs also might more often break [6] or interfere with foraging [4]. Thus, opposing selection may have begun moving maxillary overhangs back toward more intermediate values in cliff swallows.

Increased maxillary overhangs would be particularly beneficial in larger colonies, where more parasites occur [11, 13]. Given that cliff swallows have a genetically based preference and phenotypic specialization for certain colony sizes [26], we thus predicted that cliff swallows in larger colonies should have more pronounced overhangs. However, we found the opposite pattern, with larger-colony phenotypes averaging smaller overhangs (Fig 4). This trend, although statistically significant, was not particularly strong. Possibly the effect of year obscured a colony-size effect through year-based colony sampling biases (e.g., larger colonies overrepresented in the earlier years), although we found no significant statistical interaction between year and colony size (*F*_1,900_ = 0.39, *P* = 0.53) in predicting the extent of the overhang. The lack of a positive colony-size effect on maxillary overhang is not consistent with it being a genetic response by the more social phenotypes [26, 27] to the challenge of greater parasitism in the larger colonies. No information is available on the heritability of the maxillary overhang for any species [9], which complicates interpretation of empirical patterns (Figs 3 and 4) as reflecting selection in general.

Has an increase in maxillary overhang led to reduced infestations of cliff swallow parasites over time? We do not know about lice: 9.5% of free-living cliff swallows sampled in one year (1992) had one or more amblyceran lice [11], but lice have not been quantified in any other years. Our data on fleas show a reduction of about 50% among those found on nestling cliff swallows between the 1980’s and the 2010’s. Assuming those on nestlings reflect generally the level of flea parasitism on adults, it seems likely that flea parasitism has been reduced over the last 35 years. That this reduction was concurrent with the increase in the extent of the birds’ overhangs might indicate that the maxillary overhang is an adaptation to ameliorate the cost of fleas. We note that the overall reduction in flea parasitism was quantitatively small, but fleas counted on nestlings is merely an index of overall parasitism in a nest [11].

Based on collected nests where all bugs in a nest were counted, swallow bug parasitism per cliff swallow nest has not changed significantly over the past 35 years [18]; however, in 2020–21 birds with perceptible overhangs had significantly fewer bugs on the outsides of their nests [another index of parasitism; 32], suggesting the maxillary overhang is effective in controlling bugs to some extent. Had the birds not developed greater overhangs over time, the average number of bugs might be greater now than in the 1980’s. Cliff swallows have developed a greater tolerance to the effects of swallow bugs over the last 30 years [18], and their not being as negatively affected by bugs now as in the 1980’s might partly explain why the increased numbers of bugs in nests of birds without perceptible overhangs did not lead to differences in reproductive success (Fig 5). Another possibility is that our inability to distinguish overhangs of 0 from 1 under field conditions meant that having to combine nests from these two groups of birds obscured relevant variation in nesting success among them.

The predictions of this study are based on the maxillary overhang being effective in controlling flea and swallow bug parasites of cliff swallows. Without experimental studies, we do not know whether cliff swallow fleas are controlled by preening. The presence or absence of fleas did not affect the extent of preening in great tits [*Parus major*; 36] or blue tits (*P*. *caeruleus*; [37]), but blue tits rifle through nest materials, during which they may kill and/or swallow fleas in the nest [37], and a longer beak overhang might be beneficial in such activity. Gravid fleas crawl on cliff swallows’ feathers and are relatively slow-moving (C. R. Brown, pers. obs.), suggesting they could be dislodged by shearing action of a bird’s beak.

Both adult and nymphal swallow bugs crawl on the birds while seeking blood meals, especially at night, and nymphs in particular are susceptible to fatal injury when engorged and easily “pop” at the slightest touch. Preening and a maxillary overhang in all likelihood helps control bugs on the feathers; high levels of nocturnal preening are consistent with our hearing extensive bird movement inside nests at night when swallows do not come and go from the nests (C. R. Brown, pers. obs.). Swallow bugs stay mostly inside nests or on the substrate and do not frequently travel on cliff swallows outside of the nest; however, they will disperse on the birds’ legs [38] and then also are susceptible to preening.

However, we should note that not all ectoparasites can be controlled by a maxillary overhang. In experiments with rock pigeons, birds without beak overhangs were as successful at controlling highly mobile hippoboscid flies as were those with overhangs [39]. This result may have been because flies are relatively large and soft-bodied, at least as compared to lice for which advantages of the overhang have been demonstrated [1, 6]. Without experimental studies, we do not know if this situation applies to cliff swallow parasites such as fleas and swallow bugs, although when on the birds these parasites are often either attached to the skin (during feeding) in the case of bugs or slowly crawling on the feathers in the case of fleas (C. Brown, pers. obs.), so in those ways they may be unlike hippoboscid flies.

In conclusion, the intraspecific variation in the extent of the maxillary overhang in cliff swallows was partly consistent with it having a functional role in combatting ectoparasites. The increase over time (up to a point) as the birds were exposed to more parasites, the temporal reduction in fleas, and our observing fewer swallow bugs on the outsides of nests where birds had perceptible overhangs all suggested an anti-parasite role for this morphological trait. On the other hand, birds exposed to more parasites in larger colonies did not have greater overhangs than swallows in small colonies, and nesting success did not vary with the extent of the maxillary overhang. If the hippoboscid fly results [39] apply to cliff swallow parasites, the temporal changes in the extent of the maxillary overhang we documented could reflect other factors, such as decreased wear on the beak or variation in principal food type (flying insects). We have no direct data to address these possibilities. Regardless of the overhang’s precise function, our results add to those of others [40, 41] in demonstrating that avian morphological traits can rapidly change over time.

## Supporting information

S1 Data (XLSX)

S2 Data (XLSX)
